# Disclosure and silencing: A systematic review of the literature on patterns of trauma communication in refugee families

**DOI:** 10.1177/1363461514568442

**Published:** 2015-10

**Authors:** Nina Thorup Dalgaard, Edith Montgomery

**Affiliations:** University of Copenhagen; DIGNITY – Danish Institute Against Torture

**Keywords:** disclosure, refugee families, silencing, transgenerational transmission of trauma, trauma

## Abstract

This systematic review aimed to explore the effects of different degrees of parental disclosure of traumatic material from the past on the psychological well-being of children in refugee families. A majority of studies emphasize the importance of the timing of disclosure and the manner in which it takes place, rather than the effects of open communication or silencing strategies per se. A pattern emerged in which the level of parental disclosure that promotes psychological adjustment in refugee children depends on whether the children themselves have been directly exposed to traumatic experiences, and whether the children are prepubescent or older. The process of trauma disclosure is highly culturally embedded. Future research needs to address the culturally shaped variations in modulated disclosure and further explore how modulated disclosure can be facilitated in family therapy with traumatized refugee families.

## Introduction

When working with children in traumatized refugee families, a central concern is the way in which family members discuss the family's previous traumatic experiences. Should parents be encouraged to disclose traumatic material from the past to their children? And should a parental trauma history that took place before children were old enough to understand be communicated to the children? Related to these clinical dilemmas is the question of how parents should explain posttraumatic symptoms to children, who are bound to observe parental suffering. These questions have yielded divergent answers from researchers with different cultural and theoretical perspectives, and the findings are far from unequivocal. The problem first arose in the decades following World War II, as mental health professionals and researchers began reporting a number of symptoms in offspring of Holocaust survivors (Kellerman, 2001a, 2001b). A phenomenon known as the “conspiracy of silence” was reported to be the cause of much suffering within the families of Holocaust survivors (Braga, Mello, & Fiks, 2012; Fromm, 2011; Giladi & Bell, 2013; Lichtman, 1984; Sorscher & Cohen, 1997). Drawing mainly on psychodynamic theories, researchers claimed that the transmission of trauma was mediated by the lack of open communication about the past and the emotional withdrawal which was thought to characterize the survivor parent, and the transmission of trauma was seen as a result of unconscious displaced emotions (Danieli, 1998; Katz, 2003; Kellermann, 2001a; Shmotkin, Shrira, Goldberg, & Palgi, 2011). Within this theoretical understanding, parental trauma experiences are thought to become family secrets, enabling intergenerational transmission of behavioral patterns and suffering similar to the patterns seen in families in which incest and violence have been transmitted across generations (Krugman, 1987; Lesniak, 1993; Lev-Wiesel, 2006; MacFarlane & Korbin, 1983). This leads to the clinical assumption that the prevention of intergenerational transmission and family-level therapeutic change can be facilitated by the parental disclosure of family secrets.

While much research is still conducted with second and third generations of Holocaust survivors, research on non-Western refugees and survivors of other kinds of trauma, as well as their children, is now emerging. This calls for a reconsideration of the relative value of silencing versus disclosure, as many non-Western cultures have different ideals and traditions with regard to intrafamily communication (De Haene, Grietens, & Verschueren, 2010b; De Haene, Rober, Adriaenssens, & Verschueren, 2012; Rousseau & Drapeau, 1998). This observation is related to the criticism of psychological trauma interventions with traumatized non-Western refugee populations in which a central assumption is that victims of trauma need to emotionally ventilate and work through their experiences in order to avoid developing serious mental problems (Summerfield, 1999). A number of studies point to divergent effects of open communication about traumatic material from the past, and different theoretical explanations have been suggested (Abrams, 1999; De Haene, Grietens, & Verschueren, 2010a; Montgomery, 2004; Weine et al., 2004). Contributions from family systems and social constructivist perspectives enable an understanding of the effects of different communication styles as contextualized and culturally embedded, whereas the attachment paradigm emphasizes the importance of parental affective communication and parental open communication about migration-specific stressors for refugee children who have themselves been exposed to traumatic events (De Haene, Dalgaard, Montgomery, Grietens, & Verschueren, 2013). Recently, a number of studies have suggested that modulated disclosure may be associated with psychological adjustment in non-Western refugee children. The term “modulated disclosure” refers to a style of intrafamily communication in which the timing and manner of disclosure are emphasized and in which parental sensitivity to the child's cognitive and emotional needs is seen as more important than the content of what is disclosed. Based on this finding, it has been suggested that pushing disclosure in the way common in some Western psychotherapeutic settings may actually be harmful (Rousseau, Measham, & Nadeau, 2013).

Despite these diverse findings, many authors still seem to take the initial conclusions regarding the negative effects of silencing strategies for granted, although there have been surprisingly few studies in which communication patterns within non-Western refugee populations are explored empirically. The aim of this systematic review, therefore, was to summarize findings on the effects of different styles of intrafamily communication regarding traumatic experiences from the past on the mental health, psychosocial adjustment, and wellbeing of children of refugee parents in an attempt to clarify the empirical evidence addressing the controversy of disclosure versus silencing.

For the purposes of this review a refugee was defined

[A]s a person who has fled his/her social living context because of threat to the safety or integrity of themselves or family members due to any cause (e.g., war, civil conflict, disaster, oppression, or persecution that is explicitly or implicitly sanctioned by the state). (Hollifield et al., 2002, p. 618)

Trauma was not limited to experiences leading to the development of PTSD but defined broadly “as a set of extraordinary, stressful events, directly associated with the context of war or armed conflict” as experienced subjectively by an individual (Rousseau, Mekki-Berrada, & Moreau, 2001, p. 43). Traumatic events include preflight and during flight experiences of violence, torture, imprisonment, and persecution as well as witnessing violence and the loss of or separation from family members (Boehnlein & Kinzie, 1995).

## Method

The systematic review included English language, peer-reviewed publications containing empirical observations of parental patterns of trauma communication with children in refugee families. Both, studies using qualitative and quantitative methods were included. Reviews, editorials, letters, comments, commentaries and “points of view” were excluded from the review. Furthermore, the search strategy produced a significant number of studies that dealt with nonrefugee populations, with transmission of trauma unrelated to communication style, or with intrafamily communication unrelated to trauma, all of which were also excluded.

**Figure 1.**
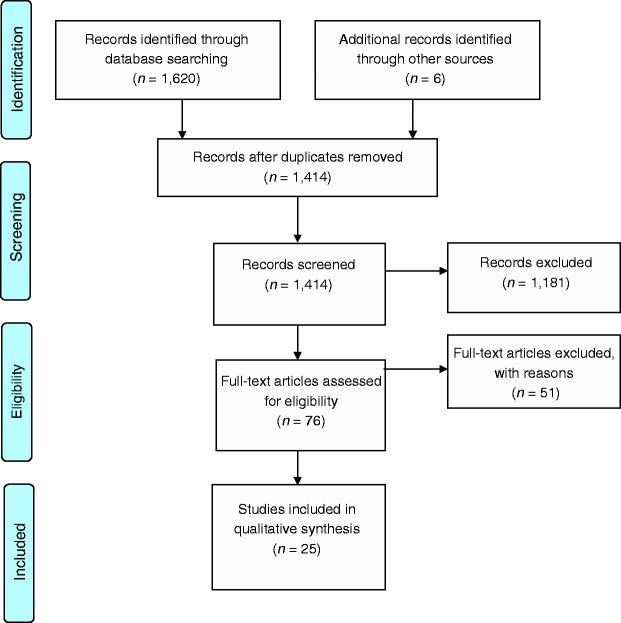
Selection of studies based on the PRISMA 2009 flow diagram (Moher, Liberati, Tetzlaff, & Altman, 2009).

Articles were identified through searches in PubMed (1999–current), PsychINFO (1806–current), PILOTS (1871–current), Scopus (1960–current), EMBASE (1974–current), Web of Science (1900–current), CINAHL (1937–current), and by checking the reference lists of articles. Initially, free-text searches were carried out in all databases using a wide range of free-text terms related to the subject. Records were screened, and the list of free-text terms that produced relevant results was limited to the terms: trauma, trans* or inter*generational, communication, disclosure or silence, refugee, family, children, linked by AND. Subsequent free-text searches were carried out in all databases using all possible combinations of these terms. After screening the resulting records, relevant MesH terms and subject headings were identified (intergenerational relations, psychiatry and psychology, communication, refugee, family, trauma) and searches using only controlled vocabulary were carried out. This did not lead to the identification of any new records. In order to identify as many records as possible, the final searches included all meaningful combinations of subject terms selected from the controlled vocabulary or thesaurus with the free-text terms listed above. Thus all searches included at least one of the central terms: refugee, intergenerational relations OR communication. This led to the identification of three additional records. Figure 1 summarizes the outcome of the search strategy.

As part of the initial screening and critical appraisal, each potential study was assessed based on judgments about relevance to the review question. All studies that met inclusion criteria were included in the review, as the nature of the review question made it difficult to predefine the appropriateness of different methodologies. During the following stages of the review process a quality assessment of each included study was undertaken, and judgments about the quality and weight of evidence were included in the discussion and interpretation of findings. The criteria used to judge quality were the extent to which the studies dealt with the question of trauma communication in a direct manner (e.g., had this as a primary focus), and the extent to which the measures used allowed for a distinction between more than just predefined categories of either disclosure or silencing (Harden & Gough, 2012).

## Results

The main results of the 25 studies identified are summarized in the Supplementary Table, which can be found online with this article (http//:tps.sagepub.com). The final selection of studies for the review included 14 quantitative studies with sample sizes of 18 or greater. Four of these studies used a mixed methods design, whereas 10 studies solely used structured measures such as standardized interviews, questionnaires, and rating scales. The remaining 11 studies were either single or multiple case studies using qualitative methods. Five studies included children of 12 years or younger, one study included children ages 12–18, and 19 studies included families with children in all age ranges or adult offspring of traumatized refugee parents.

Studies were examined for indicators of the effects of open communication and silencing strategies in different populations. A system was created in which studies were placed in three different categories: studies indicating a positive effect of open communication; studies indicating a negative effect of open communication; and studies in which a modulated approach to the disclosure of traumatic material from the past seemed to be associated with psychological adjustment in children. This category included studies in which open communication was associated with both positive and negative child outcome measures. Results are displayed in Table 1. Some studies are listed multiple times due to their design. During quality assessments for the present review, four studies were found to be unsuitable for this categorization, as the aims and designs of the studies made any conclusions about the effect of open communication or silencing too speculative. All four studies included children in all age ranges and adult offspring of refugees (Azarian-Ceccato, 2010; Boehnlein et al., 1995; Daley, 2006; Wiseman, Metzl, & Barber, 2006).

**Table 1.** Summary of findings regarding disclosure and silencing in refugee families.

Type of refugee children	Exile-born refugee children, 2nd and 3rd generation offspring of refugees	Refugee children born in home country or with direct trauma exposure
Primary finding		
Studies supporting the hypothesis that there is a need for disclosure. Disclosure of traumatic material is seen as a healing mechanism.	Braga, Mello, & Fiks, 2012^1,4^ Giladi & Bell, 2012^1,4^ Lichtman, 1994^1,4^ Montgomery et al., 1992^4^ Sorcher & Cohen, 1997^1,4^ Wiseman et al., 2002^1,4^	Almqvist & Broberg, 1997^2^ Montgomery, 2010^4^ Montgomery et al., 1992^4^
Studies indicating a negative effect of open communication. Silencing is seen as a protective factor.		Angel et al., 2001^4^ Montgomery, 1998a^4^
Studies that support the hypothesis that modulated disclosure is a protective factor. Modulated disclosure is seen as parental disclosure that is developmentally timed and carried out in a sensitive manner.	De Haene et al., 2013^2^ Lin et al., 2009^4^ Montgomery, 2004^4^ Okner & Flatherty, 1989^1,4^ Rousseau & Drapeau, 1998^4^ Rousseau et al., 2013^2^ Rowland-Klein & Dunlop, 1998^1,4^ Weine et al., 2004^4^	Bek-Pedersen & Montgomery, 2006^3^ De Haene et al., 2013^2^ De Haene et al., 2012^2^ Measham & Rousseau, 2010^2^ Montgomery, 2004^4^ Rousseau & Drapeau, 1998^4^ Rousseau & Drapeau, 1998^2^ Weine et al., 2004^4^

1 Western refugees (Holocaust survivors);

2 children 12 or younger;

3 children older than 12;

4 children in all age ranges and adult offspring of refugees.

## Discussion

The exploratory nature of the present review and the complexity of the question asked lead to several limitations. First, the search strategy only revealed studies that dealt directly with the question of trauma communication, thus studies where findings regarding trauma communication were secondary to the primary aims of the studies may not have been included. Second, the diversity of the cultural backgrounds of the families being studied may make generalizations less valid, as it is possible that different styles of communication have divergent effects in different cultural groups. Lastly, the limited sample sizes and diverse designs of the studies generally compromise the generalizability and comparability of findings.

As shown in Table 1, the majority of the studies point to modulated disclosure as a protective factor. However, this raises the question of what exactly constitutes a modulated approach to disclosing traumatic material. Measham and Rousseau suggest “that the timing and manner in which disclosure occurs may be more important than the disclosure or nondisclosure of war trauma in and of itself” (2010, p. 85). This conclusion is supported by this review, which identified several specific considerations influencing timing.

### Distinction between exile-born refugee children and children with direct trauma exposure

The distinction between exile-born refugees and children who have themselves been exposed to direct trauma seems highly relevant, as empirical findings on the effects of disclosure differ between these two populations. For exile-born refugee children, modulated disclosure appears to be more adaptive than complete avoidance of disclosure (silence and denial). This is supported by the fact that the literature search revealed no studies of exile-born refugee children in which silencing strategies were found to be protective mechanisms. With regard to children with direct trauma exposure, conclusions about the positive effects of modulated disclosure must be more tentative, as two studies suggest that silencing strategies may serve as a protective factor based on the finding that open communication about traumatic material from the past is associated with anxiety in children (Angel, Hjern, & Ingleby, 2001; Montgomery, 1998). However, both were large-scale quantitative studies that did not distinguish between different kinds of disclosure and open communication. It is likely that the studies' conclusions would have been different if a more qualitative distinction between different kinds of disclosure and open communication had been applied.

Table 1 shows that all but one of the studies suggesting that disclosure of traumatic material is healing and that silencing strategies are not adaptive once the refugee family is resettled included children of all age groups and adult offspring of traumatized parents. Almqvist and Broberg (1997) included a case study of a 4-year-old, but this child had herself experienced traumatic events. Rousseau and Drapeau (1998) found differences between children in two different age groups concerning the impact of an expressive versus a restrictive style of communication. For children 12 years of age or younger, an expressive style of communication was associated with increased anxiety, whereas a restrictive communication style was associated with internalizing symptoms in adolescent girls. Thus developmental timing seems highly important, and the findings suggest that with prepubescent children, silencing strategies may very well be adaptive in some cases. In the present review, four out of five studies including only children 12 or younger found that a modulated disclosure strategy was associated with psychological adjustment in children. Moreover, it should be noted that the only study suggesting a need for parental open communication and disclosure (Almqvist & Broberg, 1997) was a single case study. Although the conclusions seem valid based on the findings presented by the authors, the evidence pointing towards modulated disclosure is more profound. Overall, findings on developmental timing seem to favor a strategy in which the amount of disclosure and open communication is adapted to the mental capacity of the particular child and the circumstances surrounding the refugee family (De Haene et al., 2013; De Haene et al., 2012; Measham & Rousseau, 2010; Rousseau et al., 2013).

### What constitutes appropriate parental disclosure of traumatic material?

Several studies point to the positive effect of affective communication between parents and children (Bek-Pedersen & Montgomery, 2006; De Haene et al., 2013; Lichtman, 1984; Sorscher & Cohen, 1997). This affective communication can be described as sharing memories and verbally symbolizing emotional experiences (Weine et al., 2004). These findings may have implications for the manner in which traumatic material should be revealed to children, as this disclosure might be a part of a general affective style of communication within the family unit. Furthermore studies of adult offspring of traumatized parents have found negative effects of “guilt inducing communication” and “indirect communication” (Braga et al., 2012; Hollander-Goldfein, Isserman, & Goldenberg, 2011; Lichtman, 1984), which could be interpreted as examples of how information about the parental trauma history can be conveyed in a manner which is insensitive to the child's needs and should not be integrated into families' general affective style of communication.

In order to address the contradictory findings of previous studies regarding the effects of parental disclosure of traumatic experiences on the psychological adjustment of children (Montgomery, 1998; Montgomery, Krogh, Jacobsen, & Lukman, 1992), Montgomery (2004) conducted a qualitative study in which it was concluded that the disclosure of parental trauma must be carried out with congruence between the children's implicit and explicit knowledge of the family history. The study reported that in some instances parents were unaware of the fact that they were indirectly referring to the trauma history when their children were present. In other cases, children had accidentally overheard fragments of conversations between their parents, which the parents did not intend for them to hear. In both situations, there was a lack of congruence between “stories lived” and “stories told,” which left the children with only their imagination to make sense of the things they experience within the family environment, including parental posttraumatic symptoms. Thus, modulated disclosure may mean that the severity of the parents' symptoms and the parents' own inclination to discuss the trauma history may determine how much children should be told. If parents, due to their own reactions, are unable to focus on their children's needs, the influence of disclosure on the children might be negative.

### The link between trauma communication and attachment representations

Within the literature on refugee children's mental health, recent research has suggested that the potential negative impact of parental traumatization may be mediated by disruptions in attachment representations in both parents and children. These disruptions are suggested to be caused by decreased parental emotional availability (Blankers, 2013) and by damaged parental internal attachment representations (Almqvist & Broberg, 2003; De Haene et al., 2013; De Haene et al., 2010a). The theoretical explanation proposed for these findings is that the decreased emotional availability of the traumatized parents causes the child to develop an insecure or disorganized attachment style (Blankers, 2013). These findings can be seen as part of a general shift from an individual focus towards a family focus within the theoretical approach to understanding the effects of refugee traumatization (De Haene, Grietens, & Verschueren, 2007; Weine et al., 2004). Findings from the present review support this shift in focus, as they suggest that processes within the refugee family, such as trauma communication, are associated with psychological adjustment in children. Blankers (2013) studied the association between parental secure base scriptedness (a measure of attachment representations in adults) and parental sensitivity in parent–child interactions using an observational measure. As predicted, an association was found, but this association was moderated by parental PTSD symptom level and number of traumatic experiences that the parents suffered. For highly traumatized parents, higher levels of secure base scriptedness or secure attachment representations served as a key protective factor as these were associated with higher levels of parental sensitivity towards the child. It seems plausible that there may also be an association between parental attachment representations and style of communication about traumatic experiences, although no study has yet examined this. Findings from the present review could suggest that there is an association between modulated disclosure and secure attachment representations, further emphasizing the need for a more integrative approach to understanding the potential negative effect of parental traumatization on refugee children.

### Cultural variation

The studies in this review included families with a wide range of cultural backgrounds, and this probably accounts for some of the variability of the findings. Unfortunately, only two studies specifically compared trauma communication in families with different cultural backgrounds. Boehnlein et al. (1995) found that Vietnamese parents reported significantly more communication difficulties with their children than a comparable group of Cambodian parents. This finding supports the notion of cultural variation and emphasizes the need for a more culturally sensitive approach to understanding communication difficulties. Rousseau and Drapeau (1998) also found that communication difficulties between parents and children may take on different forms and expressions in different cultural groups, which further accentuates the need for increased cultural sensitivity within both research and clinical work with refugee families.

The present review of the literature seems to indicate that while some level of disclosure and open communication between parents and children may be universally beneficial, a modulated approach can be culturally embedded, and thus vary across cultures while still having the same adaptive qualities (Measham & Rousseau, 2010).

Another approach to understanding cultural variation in intrafamily trauma communication is to look at parental disclosure of traumatic material from the past as the telling of *family stories* (Bylund, 2003). Within this approach, family stories are seen as narratives that serve different functions within the family system such as the creation and maintenance of individual and collective identity. Within the parent–child dyad, the parental telling of family stories is seen as an important socialization tool. In a small exploratory study of family stories and their functions, Bylund (2003) found differences as well as similarities across ethnic groups. All families in the study identified the following major functions of family stories: entertaining, inspiring, reminiscing, teaching, passing down family history, illustrating individual traits, and relating. Yet additional functions were only identified by families with specific ethnic backgrounds and thus might be seen as unique to certain cultures. These functions were: dealing with a racist society, providing healing, learning about each other, and revealing God's protective hand (Bylund, 2003). In line with these findings, it seems reasonable to speculate that a similar pattern exists with regard to maladaptive or destructive functions of family stories, and that whether or not parental disclosure of traumatic experiences from the past is associated with a positive or negative outcome for the child might depend on what particular function the story serves.

Within the general literature on family communication, it has been proposed that family communication about traumatic or other difficult material can be evaluated along two dimensions: morally/culturally acceptable (vs. unacceptable) and functionally productive (vs. destructive; Baiocchi-Wagner, Wilson-Kratzer, & Symonds, 2012; Cupach & Spitzberg, 2007). Based on the findings from the present review this framework might prove a more useful tool for exploring the effects of parental trauma communication within refugee families than the mere identification of disclosure or silencing. This argument is supported by studies documenting cultural variation in intrafamily communication in nontraumatized families (Gudykunst & Lee, 2001; Mackey, 1988; Moriizumi, 2011; Shearman & Dumlao, 2008; Shearman, Dumlao, & Kagawa, 2011) and by studies documenting cross-cultural differences in parenting practices and parent–child interaction patterns (Gielen & Roopnarine, 2004). While a discussion of these general differences is beyond the scope of this article, it is important to keep their existence in mind when interpreting the findings from the present review.

### Clinical implications

Given the lack of clear empirical evidence, it is interesting to note that the necessity of disclosure seems to be a basic assumption in many theoretical articles on clinical work with refugee families. This review suggests that there may be a need to rethink this assumption in light of empirical evidence which suggests that “pushing disclosure in a Western way” may actually be harmful in some non-Western populations (Rousseau et al., 2013, p. 129). This conclusion is further supported by De Haene et al. (2012) who propose a more dialogical approach to family therapy with non-Western refugee families in which the level of disclosure is continuously negotiated between the therapist and the family members.

Björn, Bodén, Sydsjö, and Gustafsson (2013) suggest that positive changes after brief family therapy with refugee families may be caused by the therapy challenging the family's strategy of denial and by helping the children become more “open about their inner thoughts and feelings” (p. 276). This is supported by the empirical finding that refugee parents often underestimate the level of psychological symptoms in their children (Björn, Bodén, Sydsjö, & Gustafsson, 2011; Daley, 2006; Montgomery, 2008). Thus open communication should not necessarily include disclosure of traumatic material from the past, but must include a parental willingness to discuss the inner thoughts and feelings of their children.

## Conclusion

In the present review, a majority of the studies indicate that a modulated approach to disclosure of traumatic experiences from the past is associated with psychological adjustment in children of traumatized refugee parents. A pattern emerges in which the level of disclosure which promotes psychological adjustment in children depends on whether the children have themselves been exposed to traumatic experiences, varies between prepubescent and older children, and appears to be highly culturally embedded. A modulated approach to disclosing traumatic material is characterized by an emphasis on the timing and manner in which traumatic material is disclosed, rather than on either disclosure or silencing per se. Future research needs to address the culturally shaped variations in modulated disclosure, the way in which parental disclosure may function as the telling of family stories, and the association between modulated disclosure and attachment representations. Furthermore, research should explore how modulated disclosure can be facilitated in family therapy with traumatized refugee families.

## Supplementary Material

Supplementary material
