# Supplementary material for: Disclosure and silencing: A systematic review of the literature on patterns of trauma communication in refugee families
Source: Transcult Psychiatry. 2015 Oct;52(5):579–93. doi: 10.1177/1363461514568442 (PMC4574085; doi:10.1177/1363461514568442)
Supplement: Supplementary material [file TPS568442_suppl.pdf]

**Online Supplementary Table.** Literature review on trauma communication in refugee families.

| Study | Country of origin/relocation | General description | Outcome measures | Participants | Results |
|---|---|---|---|---|---|
| Almqvist & Broberg, 1997 | Families from the former Yugoslavia relocated in Sweden. | Theoretical article | The "World Tehnique" (or the Erica method). | $N = 1$ A case study is presented. | The authors argue for the necessity of challenging the strategies of silence and denial often seen in traumatized refugee families. |
| Angel, Hjern, & Ingleby, 2001 | Families from Bosnia-Herzegovina relocated in Sweden. | Quantitative study using structured clinical interviews and observations of the children to assess their mental health. | Each family was interviewed twice by child psychiatric staff. The interviews involved structured questions both about symptoms according to DSM-III-R and about parents' attitudes towards talking with their children about the events they had experienced. | $N = 99$ School-aged Bosnian refugee children living in Sweden. | For children who had experienced much stress, a significant pattern of associations emerged in which talking about their experiences seemed to exacerbate the negative effects. |
| Azarian-Ceccato, 2010 | Descendants of survivors of the Armenian genocide currently living in California. | Qualitative study aimed at exploring the intergenerational transmission and reverberations of trauma narratives within a community of descendants of survivors of the Armenian genocide. | Ethnographic investigation of narrative renditions of the Armenian genocide recounted in both public and private venues. | $N = 11$ Great-grandchildren of survivors of the Armenian genocide aged 12-28 years. | Describes the role of collective memory and the ways in which trauma narratives become collective stories within a community. |

(continued)

**Online Supplementary Table.** Continued

| Study | Country of origin/relocation | General description | Outcome measures | Participants | Results |
|---|---|---|---|---|---|
| Bek-Pedersen & Montgomery, 2006 | Youth from the Middle East relocated in Denmark. | Qualitative study | Open interviews, group interviews, participant observation and interviews with families. | *N* = 12 Adolescents who fled their home countries when they were 7-10 years old. | Narrative analyses show that adolescent refugees' experiences are greatly influenced by the way in which stories of the past are narrated, especially stories about the family and the internal relations and conflicts within the family. |
| Boehnlein et al., 1995 | 60 Vietnamese and 47 Cambodian refugees relocated in the United States. | Study designed to determine the extent of family problems among a clinic population of Cambodian and Vietnamese refugees. | Interviews with open-ended questions to measure family functioning in the two different cultural groups. The types of problems with children described by parents were classified into the dimensions of communication, personal behaviors, school performance, social behaviors, and antisocial behaviors. | *N* = 107 Psychiatric refugee patients. | The Vietnamese patients reported significantly more problems with their children than the Cambodians. The Vietnamese patients most commonly cited communication difficulties as their primary concern. |

(continued)

**Online Supplementary Table.** Continued.

| Study | Country of origin/relocation | General description | Outcome measures | Participants | Results |
|---|---|---|---|---|---|
| Braga, Mello, & Fiks, 2012 | Children of Holocaust survivors relocated in Brazil. | Study exploring the transgenerational transmission of trauma as perceived by adult offspring of Holocaust survivors. | The study employed a grounded theory approach based on semistructured open-ended interviews. | *N* = 15 Adult offspring of Holocaust survivors. | The study identified distinct patterns of communication between parents and children and found "open, loving, everyday communication" to be associated with resilience in children whereas "indirect communication," "fragmented discourse," and "silence, secrets, and the unsaid" were associated with increased experience of trauma [- related symptoms?] in adult children. |
| Daley, 2006 | Cambodian refugees relocated in the United States. | The study examined the relationship between perception of communication and reporting | Achenbach Child Behavior Checklist, The Youth Self Report, The Parent | *N* = 40 Second-generation Cambodian youth–parent dyads. | Children reported significantly more symptoms than their parents in |

(continued)

**Online Supplementary Table.** Continued

| Study | Country of origin/relocation | General description | Outcome measures | Participants | Results |
|---|---|---|---|---|---|
| | | of symptoms among second-generation Cambodian children and their parents using a matched-control design of clinic and community children. | Adolescent Communication Scale. | | both samples. Clinic parents reported more symptoms than community parents, but no differences were observed between children. Parents reported better communication than children, and community respondents reported better communication than their clinic counterparts. |

(continued)

**Online Supplementary Table.** Continued.

| Study | Country of origin/relocation | General description | Outcome measures | Participants | Results |
|---|---|---|---|---|---|
| De Haene, Dalgaard, Montgomery, Grietens, & Verschueren, 2013 | Refugee families from a wide range of countries relocated in Denmark ($N = 10$) or Belgium ($N = 8$). | The study examined children's narrative responses to identify migration-specific representational markers of attachment quality. | The study used a narrative attachment measure adapted for use with refugee children: The Attachment and Traumatization Story Task. | $N = 18$ children in refugee families, ages 4-9. A clinical sample of 10 children without a history of direct exposure to war or organized violence and a community sample of 8 children who fled with their families from their home countries due to war, organized violence, and persecution. | For the children who were born in the home country and had a history of direct trauma exposure, there seemed to be an association between a child being classified as "secure" and open parental communication on migration-specific stressors. |
| De Haene, Rober, Adriaenssens, & Verschueren, 2012 | Family from Caucasus relocated in Belgium. | The article describes a new dialogical approach to family therapy with refugee families. | Analysis of written notes made during and immediately after sessions by the therapist. | $N = 2$<br>A case study describing a mother and her 11-year-old son. | The authors argue that the traditional phased trauma approach should be combined with a more dialogical approach which involves the negotiation of silencing and disclosure, meaning and |

(continued)

**Online Supplementary Table.** Continued

| Study | Country of origin/relocation | General description | Outcome measures | Participants | Results |
|---|---|---|---|---|---|
| | | | | | absurdity, hope and hopelessness in a therapeutic dialogue that accepts its encounter of cultural and social difference. |
| Giladi & Bell, 2013 | Descendants of Jewish survivors of the Holocaust currently living in the United States or Canada. | Quantitative study exploring the transgenerational transmission of trauma in both second and third generation. | The Secondary Trauma Scale; family: The Family Communication Scale; and differentiation of the self: Crucible Differentiation Scale. | $N = 215$ Jewish adults (18+). Two groups of second and third generation Holocaust survivors and matched control groups for each of the first groups. | The study found that second and third generation survivors had significantly poorer family communication than their control groups (test was one-tailed with a small effect size). |
| Lichtman, 1984 | Children of Jewish Holocaust survivors relocated in Israel. | Study examining communication concerning wartime experiences in survivor families. | Questionnaires measuring parental communication, MMPI, Mosher's Forced Choice Scale of Guilt, and Hogan's Scale of Empathy. | $N = 64$ Jewish adult children of Holocaust survivors. | Parental guilt-inducing communication, early experiential awareness of the Holocaust, and indirect parental communication about the Holocaust were |

(continued)

**Online Supplementary Table.** Continued

| Study | Country of origin/relocation | General description | Outcome measures | Participants | Results |
|---|---|---|---|---|---|
| | | | | | associated with negative characteristics in the offspring. Father's willingness to talk about his experiences and the frequency of the communication were associated with positive characteristics in offspring. The study also found significant gender differences. |
| Lin, Suyemoto, & Kiang, 2009 | Participants were all born to parents who had left Cambodia between 1975 and 1990 and had relocated in the United States. | The article describes influences on intergenerational communication within refugee families about sociocultural trauma and explores how education may positively affect this communication process. | Qualitative study using a grounded theory approach. | $N = 13$ individuals aged 18+. | The article highlights ways that education may contribute to healing broken narratives within refugee families affected by war and genocide. |

(continued)

**Online Supplementary Table.** Continued

| Study | Country of origin/relocation | General description | Outcome measures | Participants | Results |
|---|---|---|---|---|---|
| Measham & Rousseau, 2010 | 15 families who had recently come to Canada from Algeria or West and Central Africa. | Qualitative study exploring the relationship between family disclosure of war trauma to children and the children's play. Creative play was hypothesized to be an indicator of children's well-being. | The Sand Tray And Storytelling Interview, parental semistructured individual interviews. | $N = 21$ children between age 6 and 12 years. | The study concludes that the timing and manner in which trauma was disclosed to children appear to be associated with the children's ability to play creatively. The study also concludes that "modulated disclosure" seems to be positively associated with the child's ability to play creatively. |
| Montgomery, 1998 | Middle Eastern refugee families seeking asylum in Denmark. | The study explored the mental health of asylum-seeking refugee children. | Structured interviews with parents. | $N = 311$ children aged 3-15 from 149 families. | Contrary to what was hypothesized, having parents who gave information about the reason for fleeing their home countries, imprisonment, and torture was found, to a certain degree, to be associated with anxiety in the children. |

(continued)

**Online Supplementary Table.** Continued.

| Study | Country of origin/relocation | General description | Outcome measures | Participants | Results |
|---|---|---|---|---|---|
| Montgomery, 2004 | Refugee families from the Middle East relocated in Denmark. | The study employed a social constructionist perspective in which a distinction was made between "stories told" (i.e., what the child is told explicitly) and "stories lived" (i.e., the child's sense of what has happened). | In-depth qualitative interviews with all family members. | *N* = 14 members of 3 different families. | The study suggests that when there are contradictions between "stories told" and "stories lived" it creates a situation of uncertainty and ambiguity, which can cause confusion, powerlessness, and action paralysis within the family unit. |
| Montgomery, 2010 | Refugee families from the Middle East relocated in Denmark. | Quantitative study aimed at assessing the long-term trajectory of psychological problems in refugee children through a 9-year follow-up study. The study divided the participants into 4 groups: (1) spared = continuing low level of symptoms; (2) reacting = increasing level of symptoms; | Parents and young refugees were interviewed separately using structured questionnaires focusing on family structure, parents and young refugees' social situation and health, the child's exposure to stressful events and indicators of social adaptation. Futhermore the young refugees | *N* = 131 youth, mean age of 15.3 years. | The study found that the variable "speaks frequently with mother about problems" was the only covariate that significantly distinguished between the spared and the traumatized groups (OR = 3.72, $p < .05$), suggesting a positive effect of |

(continued)

**Online Supplementary Table.** Continued

| Study | Country of origin/relocation | General description | Outcome measures | Participants | Results |
|---|---|---|---|---|---|
| | | (3) traumatized = continuing high symptom level; and (4) adapted = decreasing symptom level. | answered questions about intrafamily communication. | | open communication on refugee children's psychological adjustment. |
| Montgomery, Krogh, Jacobsen, & Lukman, 1992 | Families originating from Chile, Uruguay, Turkey, Afghanistan, and Iraq all relocated in Denmark. | Qualitative study exploring mental reactions and coping strategies among children of torture survivors. | Open interviews with children, open and semistructured interviews with parents, semistructured interviews including the Rutters Scale with child's teacher, home visit with observation and open interview, psychological examination including Draw-a-tree and Rorschach tests. | *N* = 11 children from 5 families with at least one of the parents having been subjected to torture. | The parents' ability and will to speak openly about their experiences in prison were found to be of utmost importance for the children's capability of handling their own traumatic experiences. Silencing strategies were common, and the authors conclude that without factual information, children are left to their own fantasies. |
| Okner & Flaherty, 1989 | 140 adult offspring of Holocaust survivors living in the United | Study exploring the effects of parental communication both in general and about the | Written questionnaires including various psychological scales and questions regarding the | *N* = 194 adult children of Holocaust survivors. | In both populations parental communication of Holocaust experiences |

(continued)

**Online Supplementary Table.** Continued

| Study | Country of origin/relocation | General description | Outcome measures | Participants | Results |
|---|---|---|---|---|---|
| | States and 54 living in Israel. | Holocaust on the psychological adjustment of adult offspring. | participants' parents' communication of Holocaust experiences. | | correlated negatively with anxiety, depression, and demoralization, and correlated positively with guilt, however in the Israeli population only the correlation with demoralization was statistically significant, implying that the extent of parental communication about the Holocaust is of less importance in Israel than in the US in determining psychological outcome in children. |

(continued)

**Online Supplementary Table.** Continued.

| Study | Country of origin/relocation | General description | Outcome measures | Participants | Results |
|---|---|---|---|---|---|
| Rousseau & Drapeau, 1998 | Children with refugee parents from either Southeast Asia or Central America all relocated in Canada. | The study explored the impact of culture on the transmission of trauma using a comparative design with children in two age groups from two different cultural groups. | The Child Behavior Checklist and questionnaires measuring the family history of trauma, parental and family characteristics and the Family Environment Scale. Furthermore semi-structured interviews were designed to cover key aspects of the intergenerational transmission of trauma including intrafamily communication. | Study 1: $N = 156$ children aged 8-12 enrolled in Montreal elementary schools. Study 2: 158 adolescents in Montreal high schools. | The disclosure of war trauma to children varied between the two cultural groups and the impact of disclosure versus nondisclosure varied between different age and cultural groups. |
| Rowland-Klein & Dunlop, 1998 | Adult offspring of Holocaust survivors relocated in Australia. | Study examining the phenomenology and intergenerational transmission of trauma focusing on the interactional process of transmission within an object relations framework. | Systematic textual analysis of semistructured interviews. | $N = 6$ adult offspring of Holocaust survivors. | 4 superordinate themes where identified in all subjects' narratives: Heightened awareness of parents' Holocaust experiences, parenting style, overidentification with parents' experiences and transmission of fear |

(continued)

**Online Supplementary Table.** Continued

| Study | Country of origin/relocation | General description | Outcome measures | Participants | Results |
|---|---|---|---|---|---|
| | | | | | and mistrust. These themes were found despite the variation in parental communication regarding the Holocaust (ranging from open disclosure to silencing). |
| Sorscher & Cohen, 1997 | All participants lived in the United States. The experimental group consisted of adult children of Holocaust survivors. | Study exploring the effect of parental Holocaust trauma on their children's Jewish identity and Holocaust-related ideation using a comparative design. | Questionnaires measuring Jewish identification, parental communication of wartime experiences, and Holocaust-related imagery. | $N = 40$ Jewish adult children of Holocaust survivors.<br>Control group:<br>$N = 38$ adult children of American Jews. | Parental communication was found to be a mediator of the transgenerational transmission of trauma. Parental affective communication was associated with ethnic identification and the authors conclude that this "underscore[s] the importance of verbally symbolizing emotional experience" (p. 499). |

(continued)

**Online Supplementary Table.** Continued.

| Study | Country of origin/relocation | General description | Outcome measures | Participants | Results |
|---|---|---|---|---|---|
| Rousseau & Drapeau, 1998 | Children with refugee parents from either Southeast Asia or Central America all relocated in Canada. | The study explored the impact of culture on the transmission of trauma using a comparative design with children in two age groups from two different cultural groups. | The Child Behavior Checklist and questionnaires measuring the family history of trauma, parental and trauma, parental and family characteristics and the Family Environment Scale. Furthermore semi-structured interviews were designed to cover key aspects of the intergenerational transmission of trauma including intrafamily communication. | Study 1: $N = 156$ children aged 8-12 enrolled in Montreal elementary schools.<br>Study 2: 158 adolescents in Montreal high schools. | The disclosure of war trauma to children varied between the two cultural groups and the impact of disclosure versus nondisclosure varied between different age and cultural groups. |
| Rousseau, Measham, & Nadeau, 2013 | Refugee families from Sri Lanka, South East Asia, and Nigeria relocated in Canada. | This mainly theoretical article describes ways in which trauma can be addressed in collaborative mental health care for refugee children. | Clinical assessment. | $N = 3$ The article presents 3 case studies. | The authors argue that modulated disclosure may be associated with positive factors in the children, but that "pushing disclosure in a Western way with respect to |

(continued)

**Online Supplementary Table.** Continued

| Study | Country of origin/relocation | General description | Outcome measures | Participants | Results |
|---|---|---|---|---|---|
| | | | | | trauma that is culturally taboo can be harmful" (p. 129). |
| Rowland-Klein & Dunlop, 1998 | Adult offspring of Holocaust survivors relocated in Australia. | Study examining the phenomenology and intergenerational transmission of trauma focusing on the interactional process of transmission within an object relations framework. | Systematic textual analysis of semistructured interviews. | $N=6$ adult offspring of Holocaust survivors. | 4 superordinate themes where identified in all subjects' narratives: Heightened awareness of parents' Holocaust experiences, parenting style, overidentification with parents' experiences and transmission of fear and mistrust. These themes were found despite the variation in parental communication regarding the Holocaust (ranging from open disclosure to silencing). |

(continued)

**Online Supplementary Table.** Continued.

| Study | Country of origin/relocation | General description | Outcome measures | Participants | Results |
|---|---|---|---|---|---|
| Sorscher & Cohen, 1997 | All participants lived in the United States. The experimental group consisted of adult children of Holocaust survivors. | Study exploring the effect of parental Holocaust trauma on their children's Jewish identity and Holocaust-related ideation using a comparative design. | Questionnaires measuring Jewish identification, parental communication of wartime experiences, and Holocaust-related imagery. | $N = 40$ Jewish adult children of Holocaust survivors.<br>Control group: $N = 38$ adult children of American Jews. | Parental communication was found to be a mediator of the transgenerational transmission of trauma. Parental affective communication was associated with ethnic identification and the authors conclude that this "underscore[s] the importance of verbally symbolizing emotional experience" (p. 499). |
| Weine et al., 2004 | Bosnian refugee families relocated in the United States. | Study exploring the family consequences of refugee trauma with emphasis on both risk and protective factors. | Qualitative study of field notes from multifamily support and education groups. | The study analyzed qualitative material from group meetings of 15 groups consisting of 7-8 families with 9 sessions each. The total number of | A grounded-theory model of family consequences of refugee trauma (FAMCORT) was constructed. The study concludes that family |

(continued)

**Online Supplementary Table.** Continued

| Study | Country of origin/relocation | General description | Outcome measures | Participants | Results |
|---|---|---|---|---|---|
| | | | | families included in analyses was 125. | interventions with refugees should help "parents and children to share memories in ways that are developmentally timed and build trust" (p. 159). |
| Wiseman et al., 2002 | Adult Israeli Holocaust survivor offspring (HSO) and a matched control group. Both groups of participants were randomly sampled from the Israel Population Registry. | Study exploring differences between Holocaust survivor families and a control group on measures of, interpersonal problems, intrafamily relationships, and communication. | Quantitative interviews and measures of interpersonal problems, central relationship, mental health, and parental communication of Holocaust experiences. | $N = 110$ | HSO who reported nonverbal communication with little information about their mothers' trauma endorsed more interpersonal distress than HSO who experienced informative verbal communication, and less affiliation than either HSO who experienced informative verbal communication or non-HSO. |

(continued)

**Online Supplementary Table.** Continued.

| Study | Country of origin/relocation | General description | Outcome measures | Participants | Results |
|---|---|---|---|---|---|
| Wiseman, Metzl, & Barber, 2006 | Israeli-born Jewish men and women whose parents migrated to Israel from Eastern European countries after 1945. | The study focused on the familial communication patterns of the parents' trauma as a context for understanding the children of survivors' experiences of anger and guilt in their interactions with their parents. | Both quantitative and qualitative measures: Relationship Anecdotes Paradigm (RAP), interviews, and core conflictual relationship themes standard categories. | $N = 52$ adult children of Holocaust survivors. | Results suggest that there is a positive association between perceiving the parent as controlling and feeling angry and between feeling guilt and perceiving the parents as vulnerable. |
